# Transformer and Adaptive Threshold Sliding Window for Improving Violence Detection in Videos

**DOI:** 10.3390/s24165429

**Published:** 2024-08-22

**Authors:** Fernando J. Rendón-Segador, Juan A. Álvarez-García, Luis M. Soria-Morillo

**Affiliations:** Departamento de Lenguajes y Sistemas Informáticos, Universidad de Sevilla, 41012 Sevilla, Spain; frendon@us.es (F.J.R.-S.); jaalvarez@us.es (J.A.Á.-G.)

**Keywords:** deep learning, sliding window, transformer, violence detection, adaptive threshold

## Abstract

This paper presents a comprehensive approach to detect violent events in videos by combining CrimeNet, a Vision Transformer (ViT) model with structured neural learning and adversarial regularization, with an adaptive threshold sliding window model based on the Transformer architecture. CrimeNet demonstrates exceptional performance on all datasets (XD-Violence, UCF-Crime, NTU-CCTV Fights, UBI-Fights, Real Life Violence Situations, MediEval, RWF-2000, Hockey Fights, Violent Flows, Surveillance Camera Fights, and Movies Fight), achieving high AUC ROC and AUC PR values (up to 99% and 100%, respectively). However, the generalization of CrimeNet to cross-dataset experiments posed some problems, resulting in a 20–30% decrease in performance, for instance, training in UCF-Crime and testing in XD-Violence resulted in 70.20% in AUC ROC. The sliding window model with adaptive thresholding effectively solves these problems by automatically adjusting the violence detection threshold, resulting in a substantial improvement in detection accuracy. By applying the sliding window model as post-processing to CrimeNet results, we were able to improve detection accuracy by 10% to 15% in cross-dataset experiments. Future lines of research include improving generalization, addressing data imbalance, exploring multimodal representations, testing in real-world applications, and extending the approach to complex human interactions.

## 1. Introduction

Detecting violent events in videos is a critical endeavour within the realms of security, surveillance, and multimedia content analysis (https://english.elpais.com/economy-and-business/2024-01-29/the-horrors-experienced-by-meta-moderators-i-didnt-know-what-humans-are-capable-of.html, accessed on 22 May 2024) [1,2,3]. The ability to accurately and efficiently identify violent acts in audiovisual material not only promotes public safety, but also finds applications in fields as diverse as human rights protection, sports event monitoring, and media monitoring [4,5]. However, this task presents a significant challenge due to the presence of false positives and negatives in existing detection systems. This problem is addressed in various studies with different approaches to its solution [6,7].

In this context, the paper proposes a novel approach to mitigate false positives and negatives in the detection of violent events in videos by focusing on its application in the CrimeNet [8] model, with the aim of reducing the number of false positives and negatives of said model in cross-dataset experiments (training on one dataset and evaluating on a different one). Although CrimeNet performs excellently on all datasets it has trained on, its performance drops significantly in all cross-dataset experiments. Our approach is based on the application of an auxiliary adaptive threshold sliding window deep learning model designed to contextualize and verify the actual presence of violent events in video sequences. The adaptive threshold sliding window, which shifts over time, improved the accuracy of the detection process in cross-dataset experiments by 10% to 15%.

The innovation of our approach lies in the integration of an auxiliary adaptive threshold sliding window model into the main detection model. This auxiliary model is trained to provide additional confirmation before issuing a final classification, thereby significantly reducing false positive and negative rates. Through this approach, our goal is to improve the overall accuracy of violent event detection systems in videos. The main contributions are as follows:

- Development of a Transformer-based model designed to function as a sliding window, effectively reducing both false positives and false negatives within a binary prediction sequence.
- Adaptive learning capability within the model to dynamically assess whether the proportion of violence versus non-violence in a prediction sequence, sized according to the adaptive threshold sliding window, is indicative enough to classify the entire sequence as violent or non-violent.
- Examination of class imbalances across various datasets when analyzed frame by frame, exploring the resultant challenges for the CrimeNet model, and implementation of the sliding window model as a strategy to mitigate this issue.
- Fusion of the sliding window model with the CrimeNet violent action detection model to create an integrated detection system, coupled with post-processing refinement techniques. This approach aims to enhance violent action detection in videos, thereby advancing the benchmark performance through cross-dataset experiments.

This paper is organized as follows: In Section 2, we review related work and datasets, and highlight the need to address the problem of false positives and negatives. In Section 3, we present, in detail, our adaptive threshold sliding window approach and the methodology we followed in its development, highlighting the most important details. Then, in Section 4, we discuss the experiments performed to validate the effectiveness of our model and the expected results. In Section 5, we present and discuss the results obtained by our model and perform a series of comparisons. Finally, in Section 6, we conclude with a summary of the findings and a discussion of the implications and future applications of our approach.

## 2. Background

### 2.1. Benchmarks in the Detection of Violent Actions in Videos

This section highlights key datasets utilized at the forefront of video violence detection research. Table 1 shows a comparative study of the different datasets covered by state-of-the-art violence detection in videos.

The study delves into multiple datasets concerning the identification of violent occurrences in videos. In particular, the XD-Violence dataset stands out, featuring 4754 untrimmed videos totaling 217 h, characterized by subtle audio cues and seven realistic anomalies. Additionally, the Hockey Fights dataset contributes 1000 violent and 1000 non-violent clips sourced from NHL field hockey games. Furthermore, the Movies Fights Detection dataset offers 200 clips from action movies, encompassing both violent and non-violent sequences. Other datasets such as Violent Flows, Real Life Violence Situations, Mediaeval-2013-VSD benchmark, RWF 2000, NTU CCTV Fights, UBI Fights, Surveillance Camera Fights, and UCF Crime datasets are also incorporated, each serving specific research objectives within the realm of video violence detection.

### 2.2. CrimeNet: A Vision Transformer Model for Video Violence Detection

CrimeNet is a Vision Transformer (ViT) model specially designed for video violence detection [8]. It uses a 16-block Transformer architecture to process sequences of video frames represented through optical flow. In addition, CrimeNet incorporates structured neural learning (NSL) with adversarial regularization to generate a structured signal that enhances its violence detection capability.

CrimeNet’s architecture is composed of the following key elements:

- Video Sequence Input via Optical Flow: Takes the dense optical flow of video frames individually as input. Each frame is fragmented into several equal-sized images called patches, and these patches are embedded into a vector via an Embedding layer. This patch-based representation allows the model to capture local and global details in the video frames.
- Sixteen-Block Transformer Encoder: A Transformer encoder consisting of 16 blocks. These blocks are responsible for processing information over time and capturing the complex relationships between video frames. The depth of the architecture allows for a hierarchical representation of visual and motion features.
- Neural Structured Learning (NSL) with Adversarial Regularization: Uses NSL [20] with adversarial regularization to generate a structured signal. This structured signal incorporates prior knowledge into the model, such as motion patterns, textures, and visual features relevant to violence detection. Adversarial regularization improves the quality of the structured signal by training the model to discriminate between real and generated signals.
- Output and Classification: The output is a binary classification that determines whether a video fragment contains violence or not. The model learns to perform this classification during training on the datasets mentioned above.

CrimeNet is designed to identify violence in videos by examining visual and motion patterns extracted from optical flow data. By analyzing temporal relationships and utilizing NSL with adversarial regularization to create a structured signal, CrimeNet can detect subtle cues associated with violence. These cues include sudden changes in motion, aggressive gestures, and changes in lighting conditions.

### 2.3. Problem of False Positives and Negatives in the Detection of Violent Events in Videos

Detecting violent events in videos stands as a pivotal challenge within the realms of computer vision and deep learning. With the surge in online video-sharing platforms, there arises a heightened demand for automated tools capable of discerning and categorizing pertinent events within multimedia content. However, this effort is fraught with the inherent risk of producing false positives and negatives, potentially undermining the efficacy and practical utility of detection systems.

Table 2 shows a summary of the state of the art, with an emphasis on the two best-performing proposals on the most popular datasets in the field of violent action detection in videos.

Given the state of the art, it is worth highlighting the excellent results obtained by the EfficientNet CNN + TSE Block model [21], a model based on convolutional neural networks to which temporal attention modules called Temporal Squeeze-and-Excitation, or TSE, blocks are added, obtaining results above 90% in all the datasets on which it is tested.

Furthermore, noteworthy are the models that combine convolutional neural networks with Transformer models or with attention mechanisms, the basis of the Transformer models. The DeVTrV2 CNN + Transformer [27] model achieves over 90% on complex datasets such as NTU CCTV Fights and UBI Fights.

It is worth mentioning the models based on semi-supervised or weakly supervised learning are capable of working with small ds or with limited instances. These achieve an accuracy of over 90% for the RWF-2000 dataset [24] and over 85% for the UCF-Crime dataset [30].

Although CrimeNet is shown in the table as a superior performing model on the NTU CCTV Fights, UBI-Fights, XD-Violence, and UCF-Crime datasets, it faces a lack of accuracy in cross-dataset experiments. The CrimeNet model trains and evaluates datasets at the frame level. That is, each frame is labeled as either violent or non-violent. This results in a significant data imbalance, since, in most of the current datasets, the number of non-violent frames is significantly higher than the number of non-violent frames. The CrimeNet model overtrains on unbalanced datasets and results in high accuracy when entering and evaluating the same dataset. However, when cross-dataset experiments are performed with CrimeNet trained on one dataset and evaluated on a different dataset, the accuracy drops by 20% to 30%. This problem generates high rates of false positives and negatives, see Table 3. From here, we face the challenge of finding a method to reduce the rate of false positives and negatives and get a model able to better generalize the concept of violent action.

To address the challenge of false positives and negatives in detecting violent events in videos, several approaches have been developed in the literature [34,35,36,37]. These methods focus on improving model accuracy and reducing classification errors. Some traditional strategies include the following:

Threshold adjustment: In many detection systems, decision thresholds can be adjusted to classify an instance as positive or negative [38]. Increasing the threshold may reduce the number of negative results, which, in turn, will reduce false negatives. However, this can also increase false positives, so finding the right balance is crucial.

Feature enhancement: Refining the features used in detection algorithms can help reduce false positives and negatives. Identifying the most relevant and significant features can improve the overall accuracy of the system [39,40].

Semi-supervised learning: Using semi-supervised learning techniques can help reduce false positives and negatives by allowing the system to learn from unlabeled positive and negative examples, which can improve discrimination between positives and negatives [41].

Sliding Window: The sliding window approach in video detection reduces false positives and negatives by analyzing video frame sequences. By adjusting its size and sliding rate, it captures events of different scales and speeds, thus enhancing the system’s accuracy in reliably identifying violent events [42].

## 3. Methodology

This section presents the methodology used to address the detection of violent events in videos. We describe the approach that combines the CrimeNet model with an adaptive threshold sliding window model to reduce false positives and negatives. While the CrimeNet model focuses on analyzing frames to determine whether they contain violent acts, the adaptive threshold sliding window model specializes in analyzing temporal sequences of events in a video to determine the amount of violence in that sequence.

Firstly, the adaptive threshold sliding window model based on the Transformer architecture was developed to analyze temporal sequences of events in videos and determine the level of violence present in each sequence. This involved designing the model architecture, including the necessary attention mechanisms or specific layers to effectively capture the temporal relationships within the video sequences.

Secondly, a synthetic dataset of binary sequences was generated. These sequences represent the labels of each frame in a sequence of frames, where ones represent violent frames and zeros represent non-violent frames. Each binary sequence is associated with a global label that determines whether the sequence is completely violent or not. For the global labeling of the sequence, an Autoencoder combined with the K-Means clustering algorithm was used. The objective of this synthetic dataset is for the model to be able to detect the sequences that contain false positives or negatives, evaluating the distribution of ones and zeros in the sequence and the label of said sequence. For example, if, in a sequence labeled as non-violent (zero), there is a smaller distribution of violence (ones), it will mean that those ones correspond to false positives in the sequence.

Thirdly, the model was trained and evaluated. The synthetic dataset was used as training and evaluation data for the model. Training was carried out for 50 epochs using an NVIDIA 2070 Super GPU. For the evaluation of the model, the AUC ROC and AUC PR metrics were used to measure the performance of detecting false positives and negatives in violent events in videos, thus completing the methodological process.

Finally, CrimeNet was combined with the adaptive threshold sliding window model. The model collected sequences of predictions from CrimeNet in binary format and determined whether these predictions contained false positives or negatives for later correction. A series of cross-dataset experiments was performed on the entire system. In addition, two comparisons were performed: one using an empirical sliding window model instead of the adaptive threshold model and another using the K-Means algorithm directly instead of the adaptive threshold model. These experiments will evaluate the performance of the combined approach and compare it with alternative methods to determine the effectiveness of the proposed model in detecting violent events in videos.

## 4. Resolution Methods

### 4.1. Adaptive Threshold Sliding Window Model

**Figure 1:**
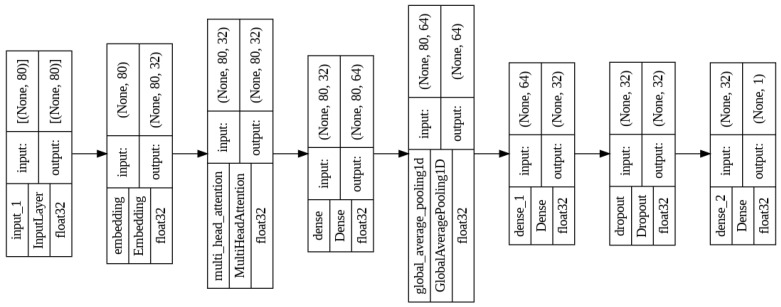
Adaptive threshold sliding window model architecture. It uses an Embedding layer together with a MultiHead Attention layer following the philosophy of the Transformer models. This architecture takes advantage of the strengths of the Transformer models to establish relationships between the different elements of a sequence of data in a one-dimensional vector.

The adaptive threshold sliding window model is built upon attention mechanisms specific to the Transformer architecture Figure 1. This model emphasizes the analysis of temporal sequences of events, using an attention structure to capture enduring relationships among sequence elements, a crucial aspect for discerning pertinent patterns within the data. The chosen loss function, binary cross-entropy, is well-suited for binary classification tasks such as violent event detection. Additionally, a synthetic dataset comprising one million binary sequences was generated for training purposes, with each sequence labeled according to its content. This synthetic dataset mirrors the process of scrutinizing a video through the adaptive threshold sliding window, enhancing the model’s robustness through simulated real-world scenarios.

### 4.2. Synthetic Data Generation, Labeling and Relationship Building

To train the adaptive thresholded sliding window model, we create a synthetic dataset representing sequences of video frames classified as violent or non-violent. This synthetic dataset is a fixed-length binary sequence of 1s and 0s representing violent or non-violent frame labels, e.g., let $S_{i}=\left[1,0,0,0,0,0,0,0,0,1,0\right]$ be a binary sequence such that $S_{i}\in D$ where *D* is the synthetic dataset. One million binary sequences are generated for the dataset. Each sequence is globally labeled as a completely violent or non-violent sequence (1, 0). One-quarter of the generated sequences are sequences in which all values are zeros (non-violent), and another quarter are sequences in which all values are ones (violent). These sequences are globally labeled with 1 for sequences in which all values are ones and with 0 for sequences in which all values are zeros. The rest of the dataset is sequences with randomly generated values. To globally label these sequences, we used an unsupervised autoencoder together with the K-Means algorithm (with the number of clusters K = 2).

**Figure 2:**
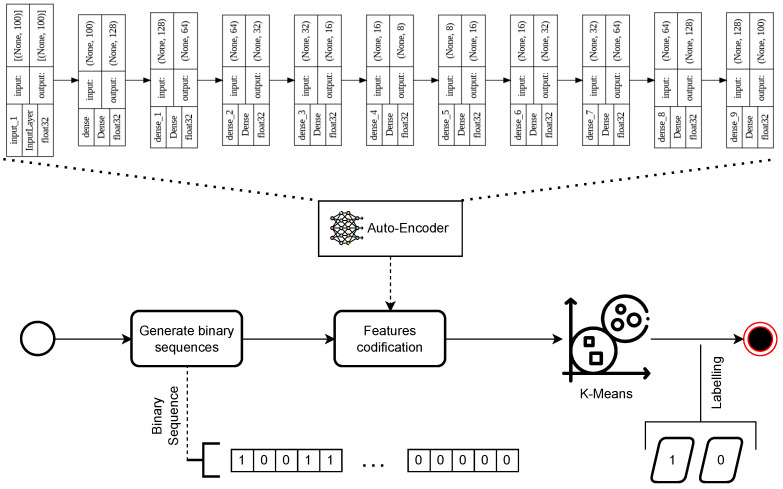
Synthetic data labeling process using autoencoder plus K-Means. The autoencoder takes the synthetically generated binary sequences as input and encodes them. From these encodings, the K-Means algorithm groups the corresponding sequences into two clusters and labels them according to the cluster to which they belong after the application of K-Means.

The autoencoder receives binary sequences as input and learns intrinsic representations of them, captures their structure, and detects the distribution of violent elements. K-Means then assigns labels to the sequences by grouping them into two categories. To test the validity of the autoencoder + K-Means combination, an ablation study was performed, in which only the K-Means algorithm was used for sequence labeling. The results can be seen in Section 5. This method dynamically identifies whether there is a “significant percentage of violence” in the binary sequence without relying on manual definitions or fixed thresholds. A significant percentage of violence refers to the distribution between zeros and ones in the binary sequence. This adaptive process ensures accurate labeling and fitting of data features, which are crucial to effectively detect violent events in videos; see Figure 2.

Once we have the synthetic dataset generated and labeled, we can move on to training and evaluating the sliding window model with an adaptive threshold.

### 4.3. Integration of CrimeNet with the Adaptive Threshold Sliding Window Model

**Figure 3:**
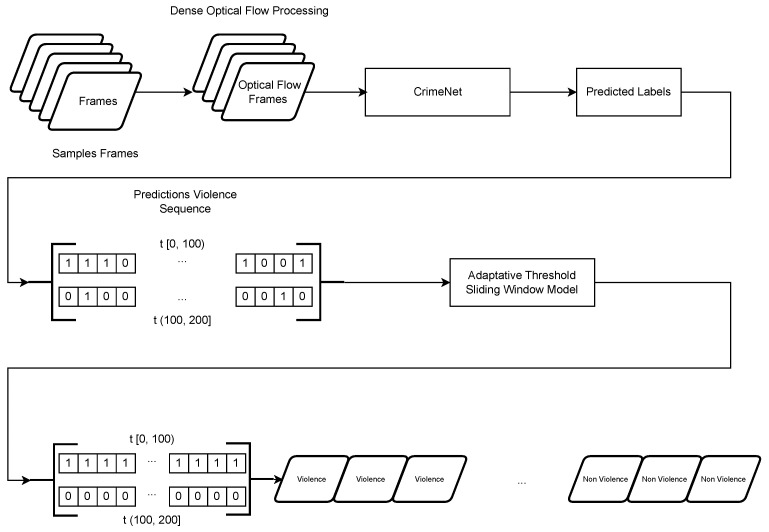
Complete system that integrates CrimeNet with the adaptive threshold sliding window model to correct false positives and negatives that may be generated by CrimeNet.

In the final phase of our approach, the adaptive threshold sliding window model, designed to enhance the outcomes of the CrimeNet model, was incorporated into a unified system for detecting violent events in videos. This system is structured into two distinct stages. See Figure 3.

In the initial stage of the system, the CrimeNet model is used. It processes the optical flow of the input video frames, generating a sequence of binary predictions. Subsequently, in the second stage, the dynamic temporal window model processes these prediction sequences. This stage harmonizes the sequences by assessing whether each one should be fully classified as violent or not. This step effectively mitigates any false positives and negatives that may have been generated by CrimeNet. Finally, the frames are labeled based on the results provided by the adaptive threshold sliding window model.

## 5. Experimental Results

In this section, we present the experimental results of our approach to detect violent events in videos using CrimeNet, as well as the Transformer-based adaptive threshold sliding window post-processing model. We evaluated their performance on the datasets described above and analyzed the impact of our approach on reducing false positives in cross-dataset experiments.

This section is subdivided into several subsections that are organized as follows:

- Section 5.1 CrimeNet Results: this subsection shows the CrimeNet results for all datasets, as well as the results of the cross-dataset experiments and the distribution of false positives and negatives across videos in those experiments.
- Section 5.2 Adaptive Threshold Sliding Window Model Results: This subsection shows the results of the adaptive threshold sliding window model with the synthetic dataset. In addition, we show the attention maps exposing how the attention mechanisms of the model work with respect to various instances of the synthetic dataset.
- Section 5.3 Results with the Adaptive Threshold Sliding Window Model in Cross-Datasets: This subsection shows the results of the cross-dataset experiments combining the adaptive threshold sliding window model with the CrimeNet model.

### 5.1. CrimeNet Results

CrimeNet demonstrates exceptional performance in detecting violent events across all datasets. It was initially trained and evaluated on the NTU CCTV Fights, UBI-Fights, XD-Violence, and UCF Crime datasets. In our experimentation, in addition to the datasets used in the original [8] publication, we tested CrimeNet on the Real Life Violence Situations, Hockey Fights, RWF-2000, Violent Flows-Crowd Violence, Surveillance Camera Fights, and Medieval-2013-VSD datasets. Then, we applied CrimeNet to the remaining datasets that achieved near-perfect results, with AUC ROC and AP metrics peaking between 99% and 100%; see Table 4.

**Figure 4:**
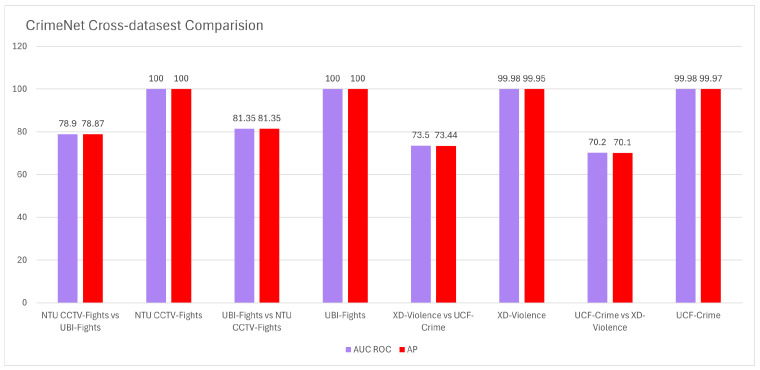
CrimeNet cross-dataset comparison with initial datasets (NTU CCTV-Fights, UBI-Fights, XD-Violence, and UCF-Crime).

To assess CrimeNet’s robustness and generalization, cross-dataset experiments were conducted. Although performance remained high when trained and evaluated on the same dataset, there was a notable decrease (20–30%) in AUC ROC and AP scores during cross-dataset experiments. See Figure 4. This suggests that CrimeNet’s performance is influenced by dataset characteristics and distribution. Furthermore, testing on movie clips outside the training datasets revealed challenges in false positive and false negative rates due to data imbalance and variability in violent event representation.

**Figure 5:**
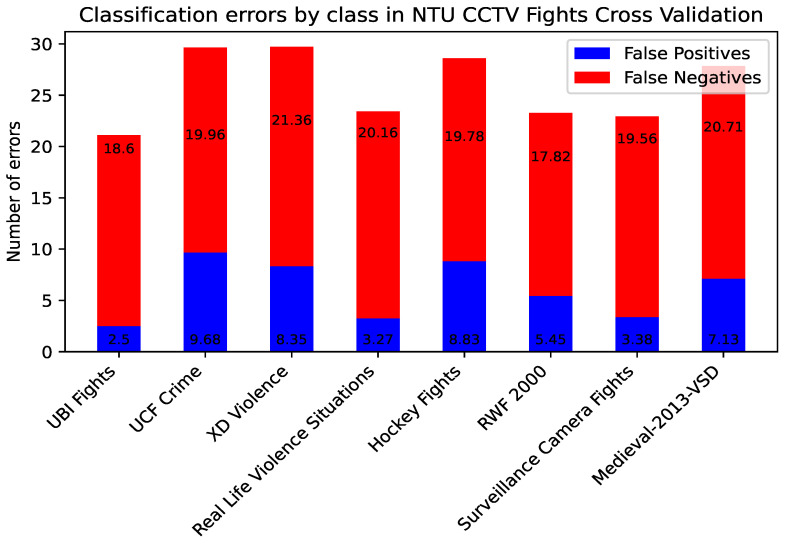
False positive and negative percentages in cross-datasets for NTU CCTV Fights.

**Figure 6:**
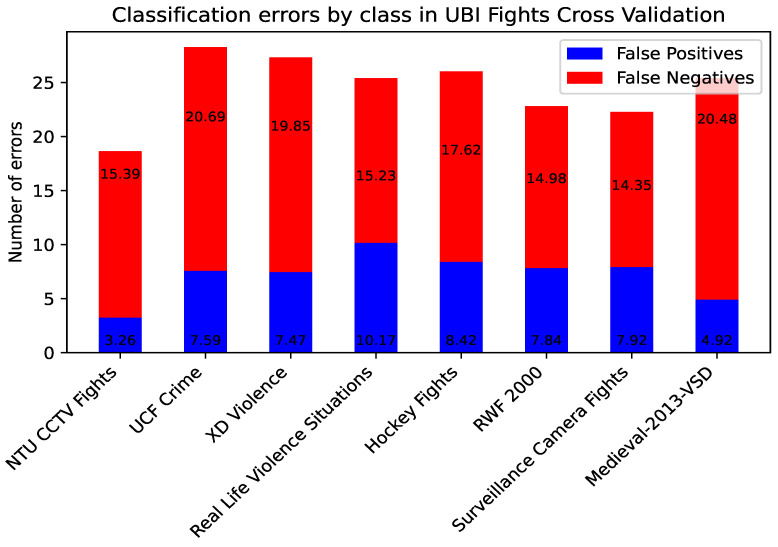
False positive and negative percentages in cross-datasets for UBI Fights.

The CrimeNet model trained with NTU CCTV Fights and UBI Fights was evaluated video by video individually with the rest of the datasets, showing that, in cross-dataset experiments, the false positives are significantly lower than the false negatives in each video. The model is biased in classifying violent actions as non-violent, as shown in Figure 5 and Figure 6. These observations underscore the need for further analysis of CrimeNet performance and its implications in real scenarios.

### 5.2. Adaptive Threshold Sliding Window Model Results

In the following, we present the results of the adaptive threshold sliding window model in our task of detecting violent events in videos to reduce false positives and negatives. This model was tested on all the datasets in which CrimeNet was also tested.

**Figure 7:**
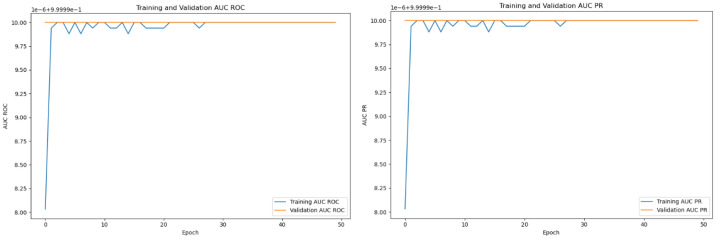
AUC ROC and AUC PR curve for the adaptive threshold sliding window model.

The performance of the Transformer Model was evaluated using key metrics, including loss function, AUC ROC, and AUC PR. The results highlight its outstanding capability. During training, the loss function shows a steady decrease, demonstrating the model’s ability to learn patterns and relationships in binary sequences. The ROC and PR curves reach 99.99% AUC, reflecting high accuracy and discrimination ability in classifying binary sequences as violent or non-violent; see Figure 7.

**Figure 8:**
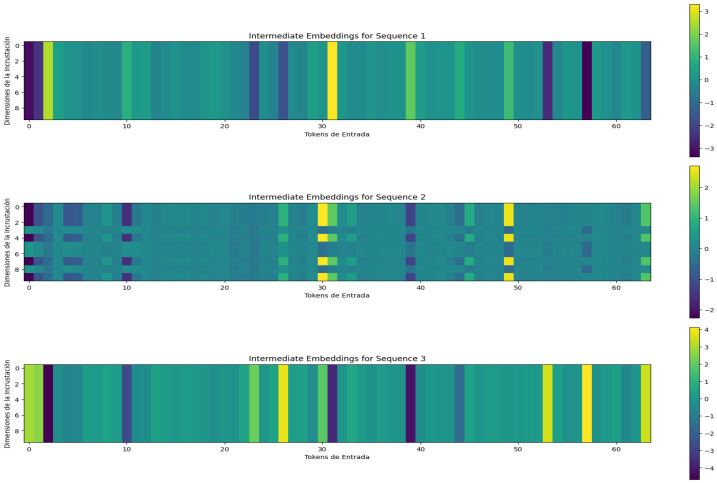
Attention maps of the adaptive threshold sliding window model for three binary sequences of length ten. The first attention map corresponds to a sequence whose values are all zeros, the second one corresponds to the sequence: [0, 0, 0, 0, 1, 1, 1, 1, 0, 1, 0] and the third one to a sequence whose values are all ones.

Figure 8 shows the attention maps for three different sequences of the adaptive threshold sliding window model. The attention maps of the sequences where all values are zeros are stable; however, the attention map of the sequence where values are zeros and ones shows how special attention is paid to the number of ones and their distribution in the sequence.

### 5.3. Results with the Adaptive Threshold Sliding Window Model in Cross-Datasets

A fundamental part of the adaptive threshold sliding window model evaluation process is the performance of cross-dataset experiments. This approach has been used to measure the robustness and generalization of the model across different datasets and partitions.

To evaluate the effectiveness of the adaptive threshold sliding window model, a cross-dataset experiment was performed in the framework of experiments using the previously trained CrimeNet model. The cross-datasets experiments process was carried out in four key stages, each with a different approach.

#### 5.3.1. Stage 1: Evaluation with Window Sizes and Manual Violence Threshold

**Figure 9:**
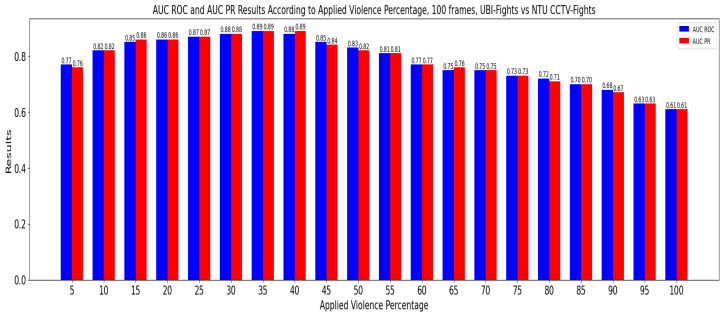
AUC ROC and AUC PR for different percentages of violence in cross-dataset of NTU CCTV Fights-UBI Fights.

**Figure 10:**
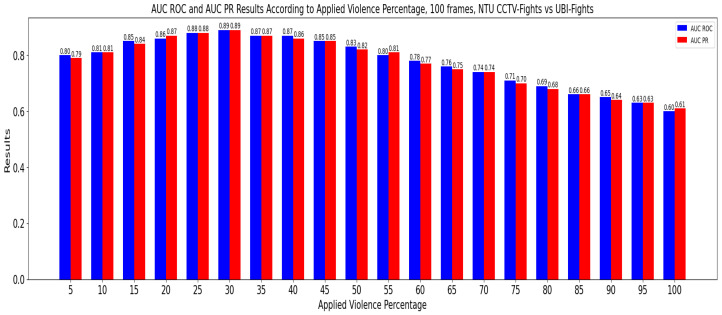
AUC ROC and AUC PR for different percentages of violence in cross-dataset of UBI Fights-NTU CCTV Fights.

In this stage, the CrimeNet model, trained first with the NTU-CCTV Fights dataset and then with the UBI-Fights dataset, was evaluated. The evaluation was performed with the remaining datasets, and a traditional sliding window was applied. Here, the values for the percentage of violence were set manually; see Figure 9 and Figure 10. For the tests, two pairs of values were set, a first pair of window size of 100 frames and a percentage of violence of 30% and a second pair of window size of 100 frames and a percentage of violence of 35%, see Table 5.

#### 5.3.2. Stage 2: Evaluation with the Adaptive Threshold Sliding Window Model

In the second stage, the results obtained in the previous stage were compared with the adaptive threshold sliding window model approach. A comparison was also made between the results with the adaptive threshold sliding window model and those obtained by CrimeNet. In this case, the same evaluation was applied to the same datasets, but instead of manually setting the values for window size and percentage of violence, the adaptive threshold sliding window model was used to dynamically determine these values; see Table 5.

The results of the evaluation of the CrimeNet model together with the post-processing of the non-adaptive threshold sliding window revealed a significant range of performance as a function of the configuration parameters. When evaluating with window sizes of 100 frames and a violence detection rate of 30%, as well as with window sizes of 100 frames and a violence detection rate of 35%, we observed that the AUC ROC and AP values ranged from 84% to 94%. These values indicate a substantial level of accuracy in detecting violent behavior in the NTU CCTV-Fights and UBI-Fights datasets.

The results shown in the table demonstrate a highly significant improvement in cross-dataset experiments, with improvements ranging from 10% to 15% when comparing CrimeNet results without the application of any post-processing to results obtained by applying the adaptive threshold sliding window model. These results are particularly notable for the AUC ROC and AP metrics.

This improvement has been consistently observed for CrimeNet trained with the datasets, NTU CCTV-Fights and UBI-Fights. Adaptive threshold sliding window adaptation, which allows the model to automatically adjust the violence detection threshold, has proven to be highly effective in improving the accuracy and efficiency of detecting violent behaviour in videos by eliminating false positives and negatives.

#### 5.3.3. Stage 3: Evaluation with CrimeNet and the Adaptive Threshold Sliding Window Model in Movies

In the third stage, a dataset consisting of fragments of different films was taken and used to perform cross-dataset experiments with pre-trained CrimeNet and with the adaptive threshold sliding window model. Four of these fragments belonging to the movies *The Butler*, *The Luck of the Logans*, and *The Battle of the Sexes* do not contain violent events; on the other hand, the movie *John Wick 2* does. A comparison was made between the results obtained with the adaptive threshold sliding window model and those obtained by CrimeNet; see Table 6.

**Figure 11:**
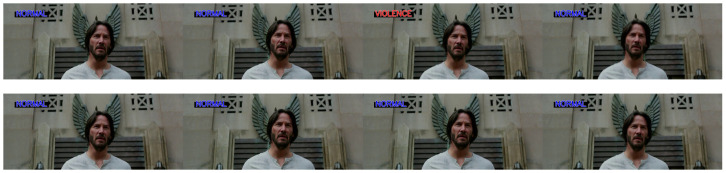
Elimination of false positives in a fragment of the movie *John Wick 2*. **Top row**: CrimeNet model predictions with one false positive. **Bottom row**: post-processing with the adaptive threshold sliding window model.

The results of the evaluation of the CrimeNet model together with post-processing with the adaptive threshold sliding window model revealed a percentage increase of between 5 and 15% in AUC ROC and AP compared to the results obtained by CrimeNet without any post-processing. For the movie *John Wick 2*, a reduction in false positives can be observed, applying the adaptive threshold sliding window model, of 8.74% for CrimeNet pre-trained with UBI Fights and 10.21% with NTU CCTV Fights. In Figure 11, we observe a fragment of this movie in which there is no violent action. The upper sequence shows one false positive and the lower sequence shows how the adaptive threshold sliding window model corrects these false positives. These results show a substantial level of reduction of false positives and negatives in cross-datasets outside of conventional datasets when applying the adaptive threshold sliding window model.

#### 5.3.4. Stage 4: Comparison between K-Means and Adaptive Threshold Sliding Window Model on a Movie Fragment

To analyze the effectiveness of synthetic data labeled using the K-Means algorithm compared to the adaptive threshold sliding window model in reducing false positives and negatives, a comparison was conducted on a specific fragment of a movie. Although both models were trained on these synthetic data, the results revealed a noticeable difference in terms of performance.

Synthetic data were labeled using K-means, creating two distinct groups to train the corresponding model. On the other hand, the adaptive threshold sliding window model was also trained on these data, but without the limitations imposed by the binary K-Means assignment.

**Figure 12:**
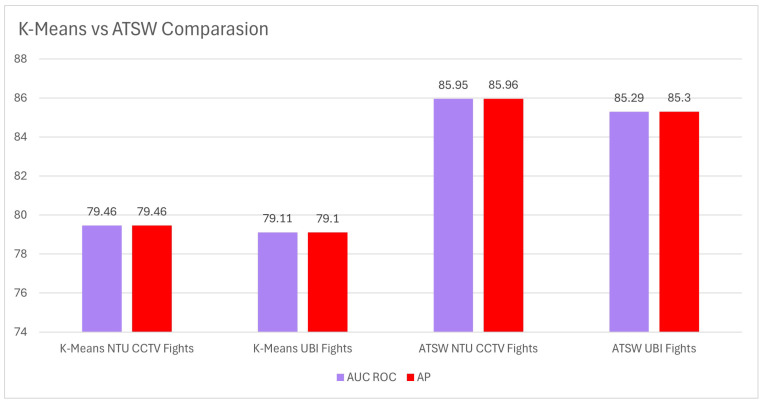
K-Means vs. adaptive threshold sliding window in *John Wick 2* cross-dataset comparison.

When evaluating this film fragment, the results showed that the K-Means model achieved an AUC ROC and AUC PR level of 79%, while the adaptive threshold sliding window model achieved a superior performance of 85%. This finding evidences that, despite being trained with the same synthetic data, the adaptive threshold sliding window model demonstrated a higher generalization ability and efficacy in reducing false positives and negatives in this specific test; see Figure 12.

This difference in performance could be attributed to the restrictive nature of the binary labeling imposed by K-Means, whereas the adaptive threshold sliding window model has the flexibility to adapt and dynamically adjust the violence detection threshold. The ability of the adaptive threshold sliding window model to more accurately capture the nuances of violent behaviors in this specific film context illustrates its robustness and versatility in event detection.

The results indicate that the adaptive threshold sliding window approach, despite relying on differently labeled synthetic data, outperforms the K-Means method in this specific task, suggesting its greater adaptability and generalizability to real-world scenarios.

## 6. Conclusions and Future Directions

In this paper, we presented a comprehensive approach for violent event detection in videos, which combines the power of CrimeNet, a Vision Transformer (ViT) model with structured neural learning (NSL) with adversarial regularization specialized for this task, with an adaptive threshold sliding window model based on the Transformer architecture to improve detection accuracy and address data imbalance issues. Our experimental results indicate that CrimeNet achieves exceptional performance in detecting violent events on specific training datasets, with high AUC ROC and AUC PR values reaching 99% and 100%. CrimeNet serves as a solid starting point in detecting violence in videos, providing a solid foundation for future research.

However, our cross-dataset experiments revealed significant challenges in generalizing CrimeNet to different datasets, where performance decreased appreciably, between 20% and 30%. We also observed that CrimeNet tended to generate false positives and negatives due to data imbalance in most of the datasets used. These challenges highlight the need to develop effective post-processing approaches that further improve the accuracy of violent event detection.

The introduction of the adaptive threshold sliding window model successfully addressed these problems, leading to a substantial improvement in the accuracy and efficiency of violent event detection. The adaptation of the adaptive threshold sliding window allowed the model to automatically adjust the violence detection threshold, resulting in a significant reduction in classification errors, both in terms of false positives and false negatives.

Future directions for this research are abundant and promising. Some key areas for expansion and refinement of this approach include the following:

- Improved Generalization. Investigate advanced techniques that will allow CrimeNet to generalize more effectively to different datasets and video scenarios, minimizing the influence of the distribution of training data. Cross-dataset experiments will further enrich the understanding of how CrimeNet performs in different contexts.
- Addressing Data Imbalance. Continue to develop approaches that mitigate the impact of data imbalance on the detection of violent events, including generating synthetic data and refining post-processing strategies. Evaluating performance in contexts with different proportions of violent events may be an additional step.
- Exploring Multimodal Representations. Investigate how combining visual data with audio information and other sensory modes can enrich representations and improve accuracy in detecting violent events. This would open the door to a more comprehensive approach to violent event detection.
- Real-World Applications. Test and adapt the proposed approach in real-world situations, such as public safety and policing, to evaluate its effectiveness in practical scenarios. Studying how CrimeNet and the adaptive threshold sliding window model behave under varying conditions can help determine their practical applicability.
- Human Interaction. Explore how this approach could be extended to the detection of violent events in more complex human interactions and scenarios, such as heated arguments and conflict situations. This would broaden the scope of the application of violent event detection.

This work represents a significant advance in the field of violent event detection in videos by presenting an effective approach that combines CrimeNet and the adaptive threshold sliding window model. Future directions offer exciting opportunities to further improve the accuracy and applicability of this technology in a variety of contexts. We hope that this work will inspire further research and advances in the detection of violent events in videos, and open the door to its implementation in real-world applications.

## Figures and Tables

**Table 1:** State of the art datasets for the detection of violence in videos.

Dataset	Multiclass	N° Classes	Time	Annotation	N° Items	Audio	FPS
Hockey Fights [9]	-	2	27 min	Video-Level	1.000	-	30
Movies Fight Detection [10]	-	2	6 min	Video-Level	200	-	30
Violent Flows—Crowd Violence [11]	-	2	15 min	Video-Level	246	-	-
Real Life Violence Situations [12]	-	2	-	Video-Level	1.000	-	-
Mediaeval-2013-VSD [13]	-	9	35.18 h	Video-Level	32.678	✓	-
RWF-2000 [14]	-	2	-	Video Level	2.000	-	5–30
NTU CCTV Fights [15]	✓	2	17.68 h	Frame-Level	1.000	✓	-
UBI Fights [16]	-	2	80 h	Frame-Level	1.000	-	30
Surveillance Camera Fights [17]	-	2	-	Frame-Level	300	-	30
XD Violence [18]	✓	7	217 h	Frame-Level	4.754	✓	24
UCF Crime [19]	✓	14	128 h	Video-Level	1.900	✓	30

**Table 2:** State-of-the-art detection of violent actions in videos. This state-of-the-art table contains the two best-performing developments for each existing benchmark in the field of video violence detection. Cross-dataset experiments are not shown in this table. * The model has been trained and tested with the corresponding dataset in this paper.

Dataset	Method	AUC ROC	Accuracy	AP
Surveillance Camera Fight	EfficientNet CNN + TSE Block [21]	-	**92.00**	-
Surveillance Camera Fight	CNN-BiLSTM + Attention [17]	-	72.00	-
Crowd Violence/Violent Flows	C3D + SVM [22]	**99.00**	**99.29**	-
Crowd Violence/Violent Flows	EfficientNet CNN + TSE Block [21]	-	98.00	-
RWF-2000	Structured Keypoint Pooling [23]	-	**93.40**	-
RWF-2000	Semi-Supervised Hard Attention [24]	-	90.04	-
RWF-2000	EfficientNet CNN + TSE Block [21]	-	92.00	-
RWF-2000	ConvNet 3D [14]	-	87.25	-
Movies Fights	EfficientNet CNN + TSE Block [21]	-	**100.00**	-
Movies Fights	ViolenceNet [25]	**100.00**	**100.00**	**100.00**
Hockey Fights	CNN+LSTM [26]	-	-	98.00
Hockey Fights	Structured Keypoint Pooling [23]	-	-	99.5
Hockey Fights	EfficientNet CNN + TSE Block [21]	-	**99.60**	-
Hockey Fights	ViolenceNet [25]	99.37	99.20	99.11
NTU CCTV Fights	DeVTrV2 CNN + Transformer [27]	-	96.00	-
NTU CCTV Fights	CrimeNet [8]	**100.00**	**100.00**	**100.00**
UBI Fights	DeVTrV2 CNN + Transformer [27]	-	91.80	-
UBI Fights	CrimeNet [8]	**100.00**	**100.00**	**100.00**
Real Life Violence Situations	DeVTr [28]	-	96.25	-
Real Life Violence Situations	DeVTrV2 CNN + Transformer [27]	-	**98.25**	-
Real Life Violence Situations	DeVTrV2 ViT [27]	-	98.00	-
UCF Crime	Magnitude-Contrastive Glance-and-Focus Network [29]	86.98	-	-
UCF Crime	BatchNorm Weakly Supervised [30]	87.24	-	-
UCF Crime	ViolenceNet * [25]	88.31	88.31	88.22
UCF Crime	CrimeNet [8]	**99.98**	**99.99**	**99.97**
XD-Violence	PEL [31]	-	-	85.59
XD-Violence	HyperVD [32]	-	-	85.67
XD-Violence	ViolenceNet * [25]	91.35	90.81	91.12
XD-Violence	CrimeNet [8]	**99.98**	**99.97**	**99.95**
Mediaeval-2013-VSD	ViolenceNet * [25]	**94.66**	**95.83**	-
Mediaeval-2013-VSD	CNN-ConvNet 3D [33]	-	78.50	-

**Table 3:** CrimeNet results in cross-dataset experiments between the different datasets on which it was evaluated.

Dataset Traning	Dataset Test	Method	AUC ROC	AP
NTU CCTV Fights	UBI Fights	CrimeNet [8]	78.90	78.87
UBI Fights	NTU CCTV Fights	CrimeNet [8]	81.35	81.35
XD-Violence	UCF Crime	CrimeNet [8]	73.50	73.44
UCF Crime	XD-Violence	CrimeNet [8]	70.20	70.10

**Table 4:** CrimeNet results for all datasets with five-cross-validation. Without adaptive threshold sliding window.

Dataset	Accuracy%	AUC ROC%	AP%
NTU CCTV Fights	100.00±0.00	100.00±0.00	100.00±0.00
UBI Fights	100.00±0.00	100.00±0.00	100.00±0.00
UCF Crime	99.99±0.01	99.98±0.01	99.97±0.02
XD-Violence	99.97±0.02	99.98±0.02	99.95±0.04
Real Life Violence Situations	100.00±0.00	100.00±0.00	100.00±0.00
Hockey Fights	100.00±0.00	100.00±0.00	100.00±0.00
RWF-2000	99.98±0.02	99.97±0.02	99.98±0.01
Violent Flows-Crowd Violence	99.97±0.03	99.95±0.03	99.96±0.02
Surveillance Camera Fights	100.00±0.00	100.00±0.00	100.00±0.00
Mediaeval-2013-VSD	99.98±0.01	99.95±0.04	99.98±0.02

**Table 5:** Cross-dataset CrimeNet, sliding window empirical parameters, and model adaptive threshold sliding window. C1 corresponds to the configuration representing the tests performed with CrimeNet without any post-processing. C2 corresponds to the configuration representing the tests performed with a static window with a pair of values (100 frames, 30% violence). C3 corresponds to the configuration representing the tests performed with a static window with a pair of values of (100 frames, 35% violence). C4 corresponds to the configuration represented by the tests performed with the adaptive threshold sliding window model. The best results are marked in blue and the second-best results are in red for each dataset.

Training Dataset	Test Dataset	AUC ROC-AP% C1	AUC ROC-AP% C2	AUC ROC-AP% C3	AUC ROC-AP% C4
NTU CCTV Fights	UBI Fights	78.9–78.87	89.05–89.11	87.68–87.55	89.32–89.3
NTU CCTV Fights	UCF Crime	70.36–70.3	88.74–88.73	87.42–87.3	90.14–90.15
NTU CCTV Fights	XD-Violence	70.29–70.29	87.69–87.72	88.15–88.15	93.45–93.37
NTU CCTV Fights	Real Life Violence Situations	76.57–76.54	88.78–88.74	90.25–90.25	93.22–93.28
NTU CCTV Fights	Hockey Fights	71.39–71.4	87.55–87.55	88.68–88.71	90.94–90.94
NTU CCTV Fights	RWF 2000	76.73–76.73	90.1–90.12	89.7–89.75	91.13–91.18
NTU CCTV Fights	Surveillance Camera Fights	77.06–77.06	89.76–89.76	91.32–91.32	92.24–92.27
NTU CCTV Fights	Mediaeval-2013-VSD	72.16–72.11	89.44–89.4	90.76–90.7	92.68–92.67
UBI Fights	NTU CCTV Fights	81.35–81.35	88.34–88.35	89.11–89.11	89.27–89.27
UBI Fights	UCF Crime	71.72–71.7	87.56–87.55	89.42–89.42	91.78–91.79
UBI Fights	XD-Violence	72.68–72.69	88.83–88.83	90.34–90.38	92.14–92.11
UBI Fights	Real Life Violence Situations	74.6–74.6	90.76–90.76	89.39–89.4	92.85–92.85
UBI Fights	Hockey Fights	73.96–73.96	89.65–89.65	88.76–88.76	90.25–90.35
UBI Fights	RWF 2000	77.18–77.13	89.75–89.73	90.59–90.6	92.73–92.83
UBI Fights	Surveillance Camera Fights	77.72–77.72	90.53–90.55	92.49–92.49	94.66–94.68
UBI Fights	Mediaeval-2013-VSD	74.6–74.62	89.93–89.93	88.79–88.82	91.87–91.94

**Table 6:** Comparison of testing in films with pre-trained CrimeNet model with or without adaptive threshold sliding window model.

Film	Dataset	Without Adaptive Threshold Sliding Window			With Adaptive Threshold Sliding Window		
		AUC ROC%	False Positive%	False Negatives%	AUC ROC%	False Positive%	False Negatives%
*The Butler*	UBI-Fights	80.33	0	19.67	85.40 (+5.07)	0	14.61 (−6.99)
*The Luck of the Logans*		81.73	0	18.26	87.19 (+5.46)	0	12.18 (−6.08)
*The Battle of the Sexes*		74.37	0	25.63	81.08 (+6.71)	0	18.91 (−6.72)
*John Wick 2*		80.75	20.56	16.92	85.29 (+4.54)	13.10 (−7.46)	17.56 (+0.64)
*The Butler*	NTU CCTV-Fights	73.28	0	26.72	81.67 (+8.39)	0	18.33 (−8.39)
*The Luck of the Logans*		76.49	0	23.51	85.27 (+8.78)	0	14.73 (−8.78)
*The Battle of the Sexes*		70.14	0	29.86	79.45 (+9.31)	0	20.55 (−9.31)
*John Wick 2*		76.70	25.23	19.88	85.95 (+9.25)	14.69 (−10.54)	12.89 (−6.99)

## Data Availability

The data used to support the findings of this study are available from the corresponding author upon request.
